# Case Report: Treatment of refractory lung disease in systemic juvenile idiopathic arthritis with cyclophosphamide and rituximab combination therapy

**DOI:** 10.3389/fimmu.2026.1798455

**Published:** 2026-05-04

**Authors:** William J. Freeman, Eileen C. Rife, Randy Q. Cron

**Affiliations:** 1 Department of General Pediatrics, Hofstra Northwell School of Medicine, Steven and Alexandra Cohen Children’s Medical Center of New York, New Hyde Park, NY, United States; 2 Division of Rheumatology, Department of Pediatrics, University of Alabama at Birmingham (UAB) Heersink School of Medicine, Children’s of Alabama, Birmingham, AL, United States

**Keywords:** case report, combination therapy, cyclophosphamide, lung disease, macrophage activation syndrome, pulmonary hypertension, rituximab, systemic juvenile idiopathic arthritis

## Abstract

**Introduction:**

Systemic juvenile idiopathic arthritis-associated lung disease (sJIA-LD) is a rare, potentially fatal complication of sJIA, often accompanied by pulmonary hypertension (PHTN) and macrophage activation syndrome (MAS). The underlying pathophysiology remains poorly understood and optimal management strategies have yet to be established. Recent retrospective data suggest that combination therapy with cyclophosphamide (CYC) and rituximab (RTX) may be effective in children with severe or life-threatening manifestations of rheumatic disease. Yet, literature on combination therapy in sJIA-LD is lacking.

**Methods:**

We describe two pediatric cases of sJIA-LD complicated by PHTN and recurrent episodes of MAS. Both patients remained refractory to conventional sJIA therapies, necessitating escalation with combination CYC–RTX.

**Results:**

Following combination CYC–RTX therapy, both patients demonstrated marked clinical improvement, including resolution of hyperferritinemia, normalization of acute phase reactants, and recovery of hepatic function profiles. Patient 1 experienced a reduction in supplemental oxygen requirement from 4 liters per minute (LPM) to 0.5 LPM nocturnally as well as improved PHTN measures: mean pulmonary arterial pressure decreased from 47 to 25 mmHg, and pulmonary vascular resistance decreased from 11.1 to 4.3 Wood units. Patient 2 experienced improved height velocity from <3rd percentile to >10th percentile and weaned off all therapies. Daily prednisone requirement for patient 1 decreased from 1 to <0.2 mg/kg/day. Chest computed tomography showed notable improvements in ground-glass opacities and septal and pleural thickening in both patients.

**Conclusion:**

Combination therapy with CYC and RTX may represent a valuable therapeutic approach in refractory sJIA complicated by lung disease, particularly in patients with concomitant MAS or PHTN.

## 1 Introduction

Management of systemic juvenile idiopathic arthritis (sJIA) complicated by macrophage activation syndrome (MAS) and/or lung disease (LD) remains a formidable clinical challenge. Current consensus guidelines from both North American and European groups recommend interleukin-1 (IL-1) and IL-6 inhibitors as first-line therapies for sJIA—with or without MAS—but acknowledge the absence of evidence-based protocols for refractory or complex cases, including those with lung involvement (1, 2). Treatment of sJIA-associated lung disease (sJIA-LD) is highly individualized and often requires escalation beyond standard biologic therapy and immunosuppressive agents (3–5). Prognosis remains guarded, with many patients experiencing persistent hypoxemia, need for ventilatory support, and limited radiographic improvement despite aggressive combination therapy (3).

Prior to the introduction of biologics, the chemotherapeutic agent cyclophosphamide (CYC) was shown to benefit severe sJIA, allowing for improved linear growth (6). Independently, CYC and rituximab (RTX) have both demonstrated efficacy in the management of systemic autoimmune rheumatic disease-associated interstitial lung disease (SARD-ILD) in adults and are recommended as first-line or adjunctive options for severe or rapidly progressive cases (7). CYC, a potent T-cell-, plasmablast-, and plasma cell-depleting agent, has been shown to improve or stabilize forced vital capacity (FVC) in SARD-ILD, though its use is limited by toxicity (7–10). RTX, a peripheral B-cell-depleting monoclonal anti-CD20 antibody, is associated with improvement or stabilization of lung function in observational studies and randomized trials (7, 11). The rationale for this combination therapy in sJIA-LD is supported by emerging immunopathologic data demonstrating upregulated type II interferon and T-cell activation networks in affected lung tissue (12) as well as clinical overlap with other SARD-ILDs responsive to lymphocyte-targeting therapies (11). Upfront combination immunosuppression is increasingly favored in severe or rapidly progressive SARD-ILD, as monotherapy is often insufficient to halt disease progression (7). A recent retrospective cohort study of 89 children by Rife et al. (13) evaluated the safety and efficacy of CYC–RTX combination therapy in non-sJIA pediatric patients with severe rheumatic disease manifestations, including pulmonary disease, and found significant glucocorticoid dose reductions with low rates of serious adverse events following this strategy.

In patients with sJIA with MAS and/or LD, strong interferon gamma (IFNγ) and IL-18 signatures are present (12, 14). IL-18 stimulates CD8-positive T cells and natural killer (NK) cells exposed to cytokines to produce IFNγ; this, in turn, signals macrophages to produce more proinflammatory cytokines, including IL-1, IL-6, and IL-18, thus perpetuating a proinflammatory cascade (14, 15). Henderson et al. (16) found that patients with acute and chronic sJIA have differences in T cell polarization; acute cases displayed Th17 polarization of regulatory T cells (Tregs), while chronic cases revealed a Th17 transcriptional signature in effector T cells. B cell depletion abolishing B cell antigen presentation reduces activated T cell phenotypes and enhances Treg number and function, which are implicated in regulating IFNγ signaling (17, 18). B cells from patients with sJIA have stronger transcription responses towards acute inflammatory responses when signaled by interferon compared to B cells from non-sJIA patients; however, the overlap in genes induced by type I and type II interferon makes their relative contributions to this profile unclear (12, 19). It stands to reason that combination CYC–RTX could offer both acute and chronic benefits via these mechanisms: IFNγ-producing lymphocyte populations could be depleted with CYC while RTX allows for the enhancement of Treg profiles, allowing a more tolerant immune system to normalize IFNγ signaling after interruption.

A number of serum biomarkers have become useful in the evaluation of sJIA-LD and MAS, particularly with respect to identifying IFNγ effects given the difficulties in obtaining reliable measurements in peripheral blood (20). CXCL9 concentrations correlate with sJIA-MAS disease activity and can help to differentiate between inflammatory LD involving and not involving IFNγ-mediated pathways (21, 22). IL-18 levels are markedly elevated in sJIA and adult-onset Still’s disease (AOSD) compared to other immune-mediated disorders, with severe elevations indicating those cases at risk for MAS and sJIA-LD (12, 23). Analysis from Schulert et al. (12) of bronchoalveolar lavage fluid collected from sJIA-LD cases disclosed elevated levels of both IL-18 and CXCL9. Soluble IL-2 receptor alpha and ferritin concentrations also tend to correlate with sJIA and MAS disease activity (24, 25). Additionally, elevated serum Krebs von den Lungen 6 (KL-6) concentrations have shown promise as a diagnostic biomarker of both sJIA-LD in children and other SARD-ILDs in children and adults (26–29).

Given the rarity and severity of sJIA-LD, we aim to describe the clinical improvements of two patients with sJIA-LD following CYC–RTX combination therapy, which may prove to be a safe and effective management strategy. To our knowledge, these represent the first reported cases of such combination therapy being used for management of sJIA-LD.

## 2 Methods

Charts of two patients with sJIA-LD treated with CYC–RTX at a single tertiary children’s hospital were retrospectively reviewed via electronic medical record. Both patients fulfilled operational definitions and classification criteria for sJIA outlined in the joint EULAR/PReS and ILAR recommendations (2, 30). Both patients fulfilled 2016 classification criteria for sJIA-MAS during their clinical courses (31). Investigations obtained at the time of patient 2’s initial presentation lacked fibrinogen and triglyceride measurements to classify MAS, though ferritin-to-erythrocyte sedimentation rate (ESR) ratio and aspartate aminotransferase (AST) measurements at the time were consistent with the diagnosis (32). Case characteristics are summarized in **Table 1**. UAB Institutional Review Board granted an exemption for reports containing three or fewer cases. Reference ranges and measurement methods for inflammatory and disease biomarkers are available in **Supplementary Table 1**. CYC–RTX dosing schedules are available in **Supplementary Table 2**. Written approval for publishing patient perspective was obtained from the family of patient 1.

**Table 1:** Select clinical characteristics of both cases of sJIA-MAS-LD managed on combination CYC–RTX.

Features	Case 1	Case 2
Demographics		
Gender	Male	Female
Race/Ethnicity	Black/African-American	Black/African-American
Medical history prior to sJIA diagnosis	Prematurity (26.4 wga) without sequelae	Severe GERD requiring fundoplication and G-tube placement at 3 months
Timeframes		
Symptom duration prior to sJIA presentation	6 months	3 weeks
Age at time of sJIA diagnosis	11 years 5 months	11 months
Age at time of LD diagnosis	11 years 8 months	2 years 7 months
Time between sJIA and LD diagnoses	2 months 29 days	1 year 7 months
Age at most recent follow-up	12 years 9 months	18 years 2 months
sJIA disease features		
Fever	Yes	Yes
Arthritis	Yes	Yes
Organomegaly	Yes	No
Lymphadenopathy	Yes	Yes
Rash	Yes	Yes
Multiple MAS episodes	Yes	Yes
Growth failure	No	Yes
Serositis	Yes	Yes
Uveitis	No	No
Raynaud	No	No
Eosinophilia	Yes	No
ANA titer	Negative	Negative
LD features		
ILD	Yes	Yes
PHTN	Definite	Probable
Digital clubbing	Yes, noted 1–2 months prior to LD diagnosis	Yes, noted 1–4 months prior to LD diagnosis
sJIA therapies (prior to/following LD diagnosis)		
GC	Yes/Yes	Yes/Yes
MTX	Yes/No	Yes/No
Anti-IL-1 agent	Anakinra/anakinra	Anakinra/anakinra
Anti-IL-6 agent	No/No	No/No
TNFi	No/No	No/No
JAKi	Tofacitinib/ruxolitinib	No/No
CNI	No/No	No/cyclosporine briefly during a 10-day hospitalization
RTX	No/Yes	No/Yes
CYC	No/Yes	No/Yes
LD therapies		
Home oxygen	Yes	No
iNO	Yes, during hospitalization	No
PDEi	Tadalafil	No
Macrolide	Yes, azithromycin	No
Diuretic	Furosemide	No
Complications		
Hypogammaglobulinemia	Yes, IVIg replacement	No
Hemorrhagic cystitis	No	No
Cytopenia	Neutropenia, CYC dose reduction and discontinuation	Thrombocytopenia, presumed secondary to subclinical MAS
Infection	Yes, CMV infection	Yes, CAP

ANA, antinuclear antibody; CAP, community-acquired pneumonia; CMV, cytomegalovirus; CNI, calcineurin inhibitor; CYC, cyclophosphamide; GC, glucocorticoid; GERD, gastroesophageal reflux disease; G-tube, gastrostomy tube; IL, interleukin; ILD, interstitial lung disease; iNO, inhaled nitric oxide; JAKi, Janus kinase inhibitor; LD, lung disease; MAS, macrophage activation syndrome; MTX, methotrexate; PDEi, phosphodiesterase inhibitor; PHTN, pulmonary hypertension; RTX, rituximab; sJIA, systemic juvenile idiopathic arthritis; TNFi, tumor necrosis factor-alpha-inhibitor; wga, weeks gestational age.

Regarding nomenclature, given the striking clinical similarity, biological characteristics, and overlapping gene-expression signatures between sJIA and AOSD, the unified term Still’s disease has been proposed to encompass both childhood- and adult-onset forms. Because this terminology has not yet been adopted universally, we continue to use the designation sJIA throughout this text while recognizing its equivalence with the term Still’s disease.

## 3 Case presentation 1

### 3.1 Patient history

An 11-year-old boy, born at 26.4 weeks’ gestation, but an otherwise healthy student-athlete, presented to the hospital with nightly fevers, joint pain and swelling, 7-pound unintentional weight loss over 1 month, and an urticarial rash evolving over 6 months. Laboratory findings were consistent with MAS, including a ferritin concentration of 14,150.8 ng/mL (normal 13.7–78.8 ng/mL), an IL-18 concentration of 144,738 pg/mL (normal ≤477 pg/mL), and elevated inflammatory markers.

Additional studies revealed elevated KL-6 at 774 U/mL (normal <500 U/mL), as well as peripheral eosinophilia (absolute eosinophil count 3,170 cells/μL; normal 30–520 cells/μL) and hypergammaglobulinemia [immunoglobulin G (IgG) 2,583 mg/dL; normal 658–1,534 mg/dL]. Targeted genetic panels for primary immunodeficiency (429 genes tested) and hemophagocytic lymphohistiocytosis (32 genes tested) returned with variants of unknown significance without any specific pathogenic variant. He was diagnosed with sJIA and MAS (31).

### 3.2 Initial management and early course

He began treatment with anakinra 2.5 mg/kg/day and methotrexate (MTX) 15 mg/m^2^/week and was discharged after 5 days.

**Figure 1:**
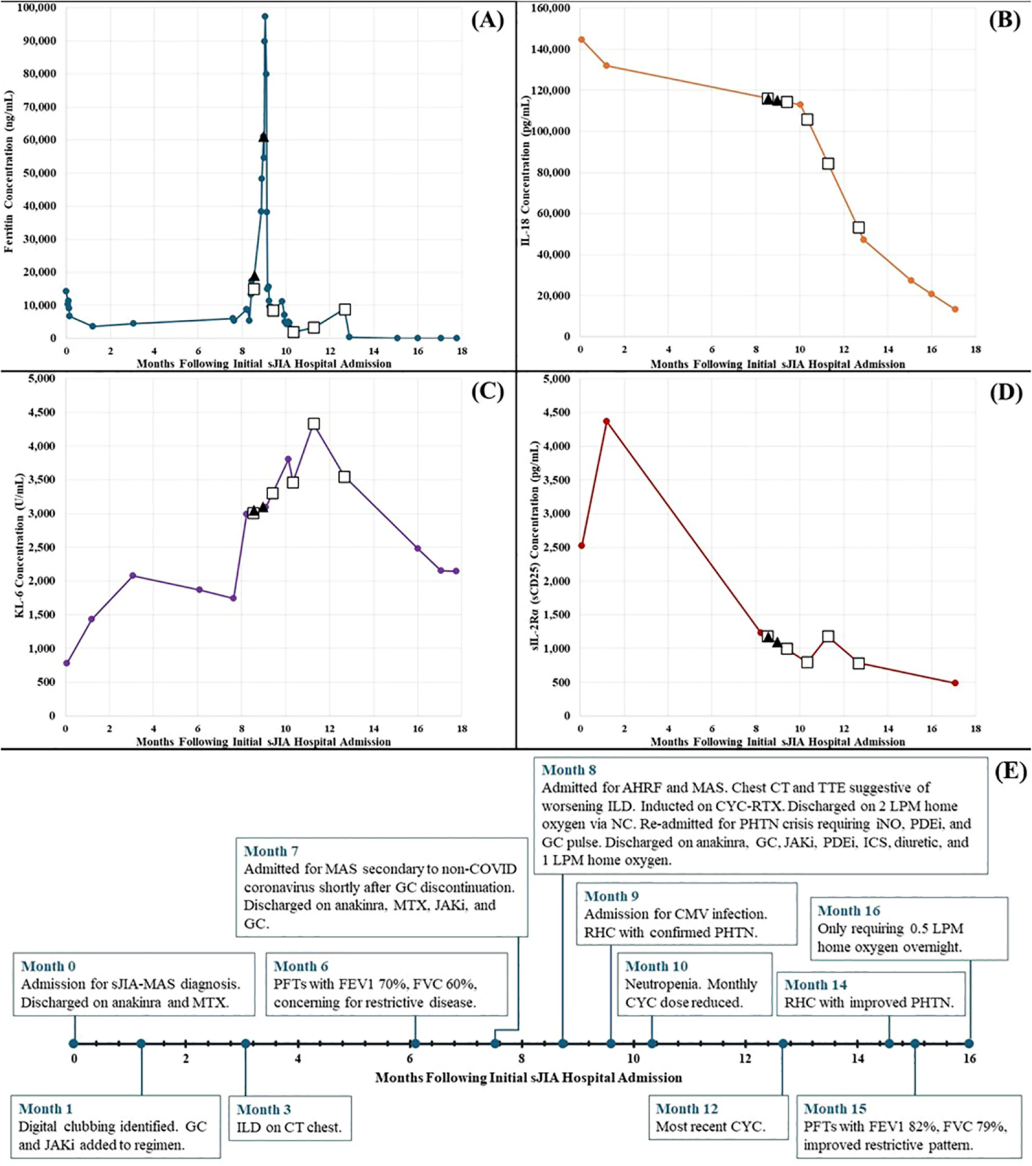
Case 1 sJIA-LD disease activity biomarkers and clinical events over time. Black triangles represent dates of rituximab infusions. White squares represent dates of cyclophosphamide infusions. **(A)** Ferritin concentration (ng/mL). **(B)** IL-18 concentration (pg/mL). **(C)** KL-6 concentration (U/mL). **(D)** sIL-2Rα (sCD25) concentration (pg/mL). **(E)** Clinical event timeline. A graph of CXCL9 concentration is available in **Supplementary Figure 1**. Refer to **Supplementary Table 3A** for inflammatory markers and disease biomarkers over time. AHRF, acute hypoxic respiratory failure; COVID, coronavirus disease 2019; CMV, cytomegalovirus; CT, computed tomography; CXCL9, chemokine (C-X-C motif) ligand 9; CYC, cyclophosphamide; FEV1, forced expiratory volume in 1 second; FVC, forced vital capacity; GC, glucocorticoid; ICS, inhaled corticosteroid; IL-18, interleukin-18; ILD, interstitial lung disease; iNO, inhaled nitric oxide; JAKi, janus kinase inhibitor; KL-6, Krebs von den Lungen-6; LPM, liters per minute; MAS, macrophage activation syndrome; MTX, methotrexate; NC, nasal cannula; PDEi, phosphodiesterase inhibitor; PFTs, pulmonary function tests; PHTN, pulmonary hypertension; RHC, right heart catheterization; RTX, rituximab; sCD25, soluble cluster of differentiation 25; sIL-2Rα, soluble interleukin-2 receptor alpha (chain); sJIA-LD, systemic juvenile idiopathic arthritis-associated lung disease; TTE, transthoracic echocardiogram.

**Figure 2:**
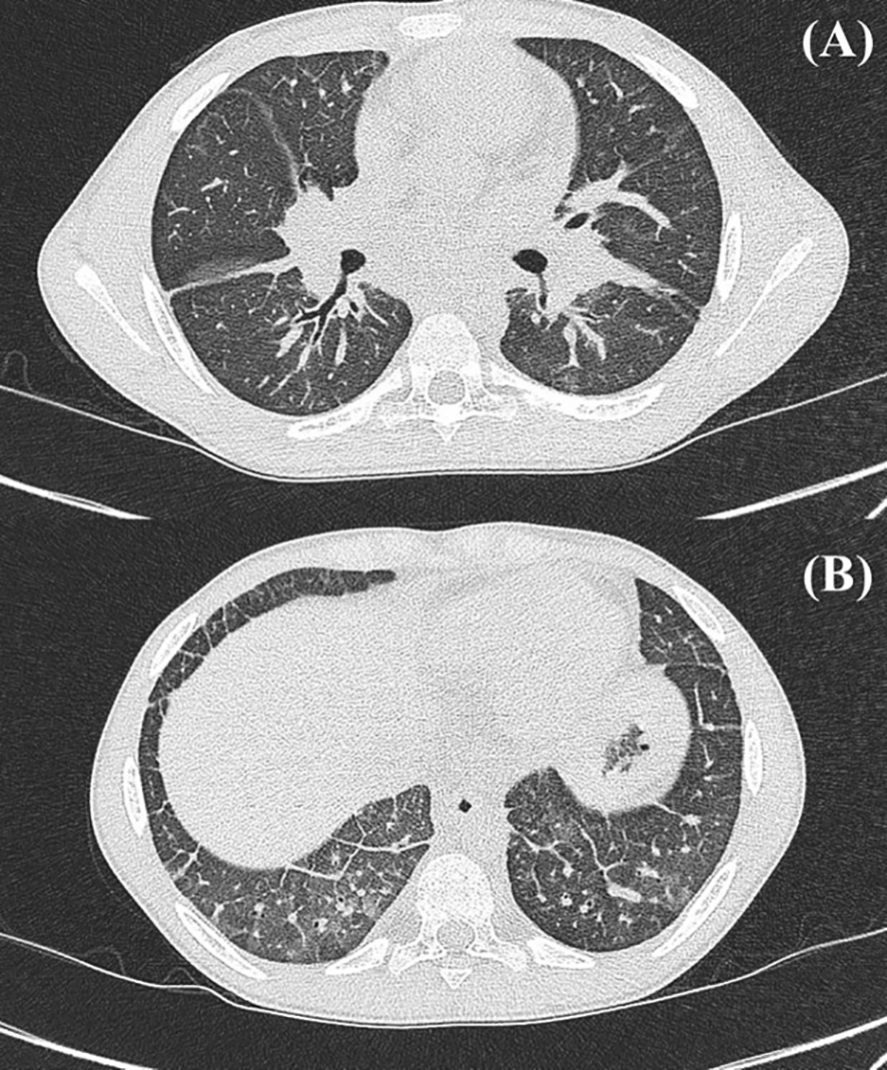
Case 1 chest computed tomography scan prior to rituximab–cyclophosphamide induction. sJIA-LD characterized by the following: **(A)** Interlobular septal and peribronchial thickening. **(B)** Bronchiectasis and peribronchial thickening in the bilateral lower lobes. Thickening most prominent in dependent portions of the lungs. sJIA-LD, systemic juvenile idiopathic arthritis-associated lung disease.

At his 1-month follow-up visit, persistent systemic inflammation prompted escalation of therapy with prednisone 1 mg/kg/day and tofacitinib 5 mg twice daily. Despite this, biomarkers remained elevated (ferritin 3,563 ng/mL; IL-18 131,901 pg/mL; KL-6 1,427 U/mL). sJIA-MAS-LD disease markers are plotted over time in **Figure 1**. With questionable clubbing on examination and a rising KL-6 concentration, a computed tomography (CT) scan of the chest was ordered, revealing subtle diffuse tubular bronchiectasis with thickening of the interlobular septa of the bilateral lower lobes (**Figure 2**). Pulmonary function tests (PFTs) revealed a forced expiratory volume in 1 second (FEV1) of 70% and an FVC of 60% predicted (FEV1:FVC 115%), consistent with restrictive and obstructive disease.

### 3.3 Disease flares and evolving pulmonary complications

He completed a 6-month prednisone taper. Shortly after discontinuation, he was hospitalized with fever, rash, arthritis, and laboratory findings consistent with MAS. He had a new oxygen requirement and repeat CT revealed worsening interlobular septal thickening and reticulation. He received pulse glucocorticoids for 3 days and was discharged on anakinra 2.2 mg/kg/day, MTX 15 mg/m^2^/week, tofacitinib 5 mg twice daily, and methylprednisolone 0.7 mg/kg/day with *Pneumocystis* and ulcer prophylaxis. He required no supplemental oxygen by discharge, and transthoracic echocardiogram (TTE) demonstrated no abnormalities.

Two weeks later, near completion of his steroid taper, he was readmitted for acute hypoxic respiratory failure (AHRF) and MAS. He required 2 liters per minute (LPM) of supplemental oxygen via nasal cannula (NC) to maintain saturations. Chest CT angiogram excluded pulmonary thromboembolism but revealed dilation of the pulmonary arteries and right heart chambers as well as cystic bronchiectasis in the bilateral upper lobes and mediastinal lymphadenopathy. TTE was concerning for moderate right ventricular hypertension [tricuspid regurgitant jet velocity (TRJV) 3.80 m/s; normal TRJV less than 2.5–2.8 m/s] with mild right ventricular hypertrophy. Despite two additional methylprednisolone pulses, oxygen dependence persisted. Bronchoscopy demonstrated inflamed, mucus-filled airways, suggestive of bronchitis and pneumonia. He was subsequently transitioned from MTX and tofacitinib to CYC 750 mg/m^2^ and RTX 750 mg/m^2^, resulting in normalization of right-sided pressures on TTE. He was discharged 14 days later on anakinra 5 mg/kg/day, prednisone 1 mg/kg/day, fluticasone, montelukast, prophylactic azithromycin (for bronchiectasis), and 2 LPM oxygen via NC. He required two assistive devices for airway clearance at time of discharge.

### 3.4 Progressive pulmonary hypertension

He was readmitted 6 days later for acute on chronic hypoxic respiratory failure requiring 4 LPM oxygen via NC. Imaging again ruled out thromboembolism but showed worsening ground-glass opacities throughout both lung fields. TTE revealed a TRJV of 4.80 m/s with dilation of the right branch and main pulmonary arteries concerning for severe pulmonary hypertension (PHTN). He was started on nitric oxide via HFNC (high flow nasal cannula) with his fraction of inspired oxygen up to 70% (normal atmospheric oxygen fraction 21%), tadalafil, and milrinone infusions. He received an additional methylprednisolone pulse, a second RTX infusion (750 mg/m^2^), and ruxolitinib 10 mg twice daily (33). A right-heart catheterization (RHC) confirmed PHTN with a pulmonary vascular resistance (PVR) of 11.1 Wood units (normal <2 Wood units), a mean pulmonary artery pressure (mPAP) of 47 mmHg (normal 10–20 mmHg), and a pulmonary capillary wedge pressure (PCWP) of 11 mmHg (normal 6–12 mmHg). He received his second CYC infusion (850 mg/m^2^) and was discharged on anakinra 5.4 mg/kg/day, ruxolitinib 10 mg twice daily, prednisone 1.5 mg/kg/day, tadalafil 40 mg daily, furosemide, fluticasone, montelukast, azithromycin, and 1 LPM oxygen via NC (in addition to scheduled outpatient CYC–RTX infusions).

### 3.5 Subsequent infections and immunosuppression-related complications

One week later, he was readmitted for respiratory distress requiring up to 4 LPM oxygen via NC. Imaging was reassuring against pulmonary thromboembolism, revealing only mild bilateral atelectasis and peribronchial thickening. TTE demonstrated moderate right ventricular hypertrophy with TRJV 3.70 m/s and mild systolic dysfunction. While BAL was considered for evaluation, given his distress and concern for exacerbated PHTN, the procedure was deferred for safety concerns. Given the uptrend of cytomegalovirus (CMV) viremia (714,900 IU/mL from undetectable 1 month prior) in an immunocompromised child with slow respiratory recovery, ganciclovir was initiated for symptomatic CMV infection prior to discharge.

At his third outpatient CYC infusion, laboratory testing revealed neutropenia [absolute neutrophil count (ANC) 1,070 cells/μL; normal 1,540–1,740 cells/μL] prompting CYC dose reduction to 750 mg/m^2^. At his fifth outpatient CYC infusion, his oxygen requirement decreased to 0.5 LPM NC, and laboratory testing revealed hypogammaglobulinemia with a quantitative IgG of 411 mg/dL and worsening neutropenia (ANC 80 cells/μL). CYC was subsequently discontinued, and he received intravenous immunoglobulin (IVIg) replacement therapy at 400 mg/kg/month for three doses with normalization of quantitative IgG levels.

### 3.6 Most recent clinical status

He was recently genotyped as HLA-DRB1*11/HLA-DRB1*15. Two months following his fifth and final CYC infusion (cumulative CYC dose 3,950 mg/m^2^), repeat RHC revealed improved pulmonary pressures: PVR 4.3 Wood units, mPAP 24 mmHg, and PCWP 13 mmHg. Repeat PFTs showed improved restrictive indices with FEV1 82%, FVC 79%, and FEV1:FVC 102% of predicted (from 70%, 60%, and 115%, respectively). He is off all airway clearance assistance devices, weaned to 0.5 LPM oxygen overnight, and down to prednisone 0.18 mg/kg/day with plans to further wean. The remainder of his therapies are as follows: anakinra 3.7 mg/kg/day, ruxolitinib 30 mg twice daily, RTX, tadalafil 40 mg daily, and furosemide. He is 12 years old, is enrolled in the seventh grade, walks 1 mile daily, and has tentative plans to return to playing American football in the coming season. He feels that his treatment process has been good and that he is getting ready to return to athletics. His mother is satisfied with his progress, stating “Treatment has been great. Prior to this therapy, I thought I was going to have to bury my child this year”.

## 4 Case presentation 2

### 4.1 Patient history

A 10-month-old girl with severe gastroesophageal reflux disease necessitating fundoplication and gastrostomy tube placement was admitted with 2 weeks of daily fever, maculopapular truncal rash, and inability to bear weight on the right lower extremity. Inflammatory markers were elevated with ferritin 2,889 ng/mL, ESR 99 mm/h (normal <15 mm/h), CRP 16.93 mg/dL (normal <0.50 mg/dL), and lactate dehydrogenase (LDH, 2,629 U/L, normal 460–1060 U/L). MRI revealed a 4-mm right hip effusion, raising concern for septic arthritis. Right hip arthrocentesis revealed cloudy blood-tinged aspirate with 66,150 WBC/μL, 85% polymorphonuclear cells, and 698,250 RBC/μL. Cultures grew coagulase-negative *Staphylococcus* in broth medium only. She began empiric treatment with cefazolin and was discharged home to complete her antibiotic course.

She was readmitted 3 days later, after encountering social barriers to antimicrobial course completion. During the admission, she experienced persistent daily fevers, generalized lymphadenopathy, rash, and arthritis of the wrists and small joints of the hands and feet. CRP and ESR remained elevated at 13.93 mg/dL and >140 mm/h, respectively. AST at this time was 57 U/L (normal 20–60 U/L). This constellation of findings expanded the differential to include sJIA and malignancy. Bone marrow biopsy was reassuring against malignancy, and she was started on prednisolone 2 mg/kg/day, naproxen, and gastroprotection for sJIA.

### 4.2 Initial management and early course

MTX 12 mg/m^2^/week and anakinra 1.6 mg/kg/day were added to her regimen. She experienced subsequent resolution of fever, rash, and arthritis as well as improved laboratory measures: ESR from 97 to 29 mm/h, CRP from 13.19 to 1.75 mg/dL. She had no further issues.

### 4.3 Pulmonary complications

Two years following initial diagnosis, she was admitted for cough, fever, and night sweats. Digital clubbing was noted, and CT chest revealed chronic changes, including extensive reticular interstitial densities and septal thickening. Given acute presentation, she was worked up for infection and found to have Gram-positive alpha-hemolytic streptococci and *Hemophilus* spp. on bronchial lavage. TTE revealed normal cardiac structure and function, a TRJV of 2.2 m/s, and an estimated right ventricular systolic pressure (RVSP) of 25 mmHg (normal 15–25 mmHg). She was discharged on anakinra 2.8 mg/kg/day, prednisolone 0.05 mg/kg/day, gastroprotection, and cefuroxime. Prednisolone was tapered shortly after discharge.

She was readmitted 1 year later for AHRF requiring 1.5 LPM oxygen supplementation. Chest radiograph revealed a new right lower lobe infiltrate, and she was prescribed antibiotics for presumed community-acquired pneumonia. Chest CT angiogram showed no evidence of thromboembolism. TTE revealed a flattened interventricular septum, dilated right atrium with a bowing interatrial septum, TRJV 4 m/s, and estimated RVSP 76 mmHg—concerning for PHTN. She had several abnormal laboratory findings including elevated aldolase (10 U/L, normal 3.4–8.6 U/L), AST (91 U/L, normal 13–26 U/L), creatine kinase (CK, 227 U/L, normal 24–175 U/L), and LDH (1,503 U/L, normal 500–920 U/L), as well as coagulopathy without MAS (ferritin 51 ng/mL; ESR 15 mm/h; CRP 2.51 mg/dL; D-dimer 3,191 ng/mL, normal 110–240 ng/mL; fibrinogen 162 mg/dL, normal 164–458 mg/dL). She was discharged after 9 days on anakinra 3.7 mg/kg/day, prednisolone 1.5 mg/kg/day, and gastroprotection without any requirement for supplemental oxygen.

### 4.4 Evolving disease

Given her severe LD and high steroid requirement, she was admitted 1 week later for induction therapy with CYC 500 mg/m^2^, RTX 750 mg/m^2^, and pulsed methylprednisolone. ANA, ENA (extractable nuclear antigen) panel, and myositis autoantibody profiles were unrevealing. TTE revealed TRJV ≤3 m/s and an estimated RVSP of 36 mmHg. She was discharged on anakinra 4.5 mg/kg/day and prednisolone 1.8 mg/kg/day. She received a second RTX infusion and six additional monthly CYC infusions with resolution of previously elevated inflammatory markers. She was weaned off prednisolone during this time.

### 4.5 Multisystemic remission and long-term course

She was admitted 8 months following CYC–RTX induction discharge for progressive respiratory symptoms and received CYC 700 mg/m^2^, pulse methylprednisolone, and two doses of RTX 750 mg/m^2^. TTE revealed normal structure and function. Her digital clubbing had improved from 4+ to 1+. Chest CT revealed improvement of interstitial markings. She was subsequently lost to follow-up for the remainder of the year, with no further hospital admissions during that period.

**Figure 3:**
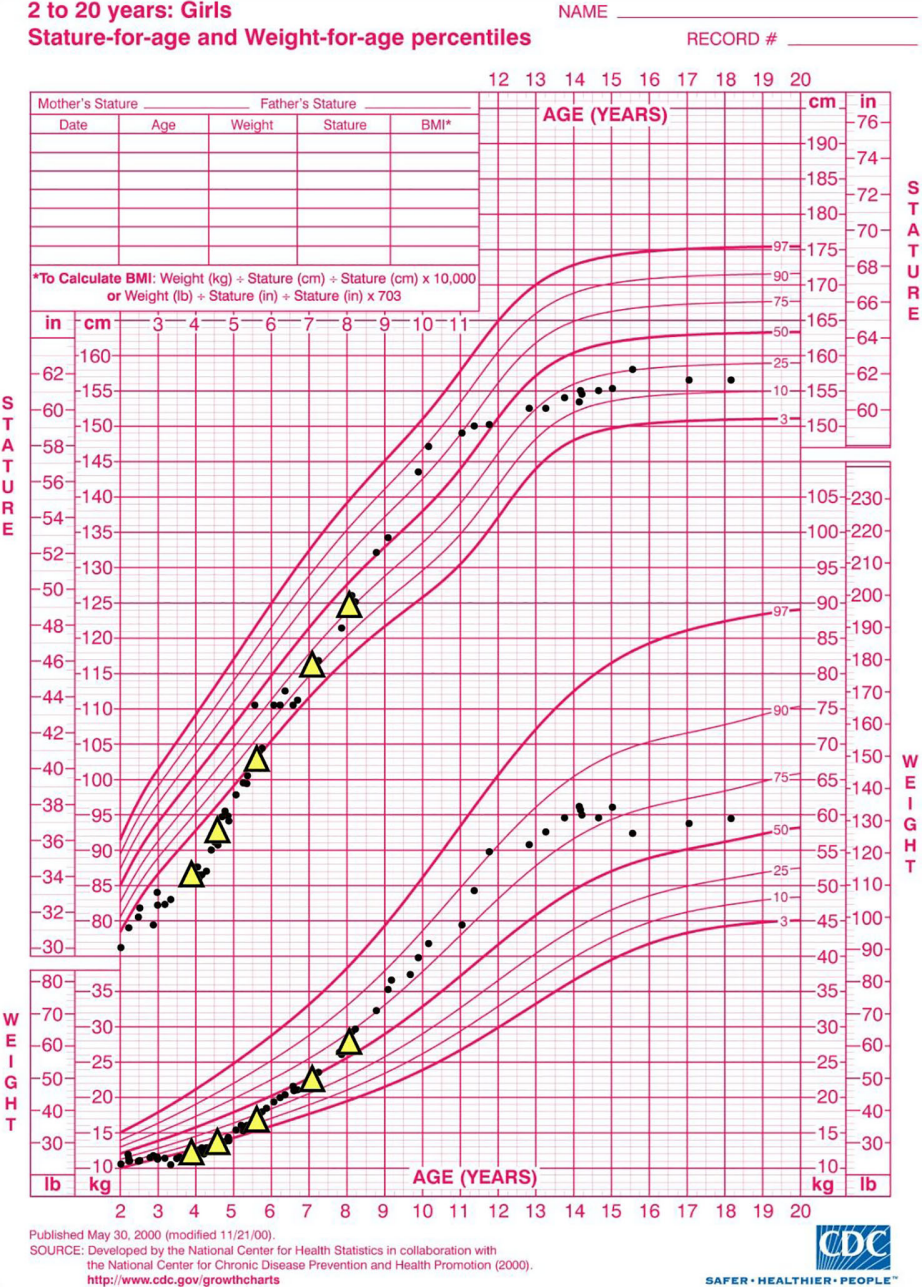
Case 2 sJIA-associated growth failure and recovery with rituximab–cyclophosphamide therapy as plotted on CDC growth curves. Yellow triangles represent dates of rituximab infusions. The patient received monthly cyclophosphamide infusions between the first and second rounds of rituximab infusions, during which time prednisolone was discontinued from 1 mg/kg/day prior to induction. Greatest improvements in height velocity occurred between years 4 and 6 of life, correlating with the first three rounds of rituximab infusions, prednisolone discontinuation, and improvements in inflammatory markers. Refer to **Supplementary Table 3B** for inflammatory marker values over time. CDC, Centers for Disease Control and Prevention; sJIA, systemic juvenile idiopathic arthritis.

After re-establishing care, she received a third cycle of CYC–RTX (CYC 700 mg/m^2^ and two doses of RTX 700 mg/m^2^, cumulative CYC dose 4,800 mg/m^2^) for recurrence of inflammation on labs, arthritis, and pulmonary signs. Her symptoms and lab abnormalities resolved 4 months thereafter and she demonstrated sustained improvement in growth velocity (**Figure 3**), prompting removal of her gastrostomy tube.

Sixteen months later, her labs revealed worsening inflammation (ESR 42 mm/h; CK 264 U/L; LDH 823 U/L), and she received a fourth cycle of RTX 700 mg/m^2^ (two infusions). She was again lost to follow-up for 2 years. After re-establishing care, she received a fifth RTX cycle (720 mg/m^2^ for two doses) in addition to anakinra 2.4 mg/kg/day.

### 4.6 Remission and recent clinical status

Given her clinical stability, she was slowly weaned off all medications, anakinra being the last. Chest CT, performed nearly 10 years following initial hospitalization, showed marked improvement with only a subtle degree of tubular bronchiectasis in lower lung fields and subtle platelike scarring in the left lung base and right middle lobe. PFTs performed 13 years following initial hospitalization showed continued improvement with FEV1 75%, FVC 76%, and FEV1:FVC 99% of predicted values (although with poor technique). She has been in medication-free remission from age 15 years through her most recent encounter at 18 years old. She was scheduled for an appointment with our pediatric to adult rheumatology transition clinic; however, she was lost to follow-up and unable to be contacted to obtain patient perspective.

## 5 Discussion

The observed clinical improvement in these cases suggests that CYC–RTX may be a viable management strategy for sJIA-LD refractory to conventional sJIA treatments, including IL-1 inhibition. Both cases demonstrated improvements in inflammatory markers, PHTN measurements, radiographic evidence of LD, and daily glucocorticoid dose on this regimen. Improvements in severe manifestations of sJIA-LD were seen shortly after CYC–RTX induction in both cases: patient 1 was discharged from the hospital for respiratory failure and pulmonary hypertensive crisis within 1 week after each of his initial CYC infusions, and patient 2 showed improvements in estimated right heart pressures on TTE within 1 month of induction. Patient 1 experienced a significant reduction in nonpharmacologic burden for management of his underlying LD following CYC–RTX, including discontinuation of airway clearance devices and reduction of home oxygen from 2 LPM continuously to 0.5 LPM nocturnally. Patient 2 experienced a significant increase in linear growth velocity during the initial three rounds of therapy and progressed as far as medication-free remission.

Nevertheless, the use of aggressive immunosuppression must be balanced against the risk of infection and other adverse effects, especially in children with prior MAS or ongoing systemic inflammation (3, 4, 7). Both patients developed cytopenias (neutropenia and thrombocytopenia, respectively) and infections (reactivated CMV and community-acquired pneumonia, respectively) following induction on CYC–RTX, attributable to the combination of their underlying diseases and their therapies.

Limitations to generalizability exist in this report. Patient 1 displayed a more classic phenotype of sJIA-LD, while patient 2’s disease course involved concurrent myositis and nephrotic manifestations over years of her life. These features may have proven more responsive to the RTX monotherapy she received in her later years of treatment. Patient 1 displayed eosinophilia while patient 2 did not. Additionally, patient 1 received JAKi therapies shortly before and after diagnosis with sJIA-LD around the time of his development and resolution of pulmonary hypertensive crisis, which could confound his clinical and laboratory improvements (34, 35).

While improvements in severe manifestations of sJIA-LD were seen in both patients shortly following CYC–RTX induction, time to chronic LD improvement was significantly delayed following initial infusions. This could reflect that the LD, like the arthritis, seen in sJIA transitions from an innate to an adaptive immune dysregulation that yields damage requiring different therapeutic strategies (36, 37). The heterogeneity of sJIA-LD, the lack of standardized treatment protocols, and the absence of controlled pediatric trials underscore the need for collaborative research to better define optimal management strategies (3, 4, 38, 39).

In summary, sJIA-LD is a rare but serious complication of sJIA, with evidence suggesting a complex interplay between biologic therapy, underlying disease severity, genetic risk factors, and immune dysregulation. These cases support the consideration of combination therapy with CYC–RTX in cases of refractory sJIA-LD, particularly those complicated by PHTN and who have progressed despite IL-1 inhibition. These cases also highlight the need for individualized, multidisciplinary care and the development of evidence-based guidelines for this high-risk population.

## Data Availability

The original contributions presented in the study are included in the article/**Supplementary Material**. Further inquiries can be directed to the corresponding author.
