# Supplementary figures and images for: Case Report: Treatment of refractory lung disease in systemic juvenile idiopathic arthritis with cyclophosphamide and rituximab combination therapy
Source: Front Immunol. 2026 May 4;17:1798455. doi: 10.3389/fimmu.2026.1798455 (PMC13180542; doi:10.3389/fimmu.2026.1798455)

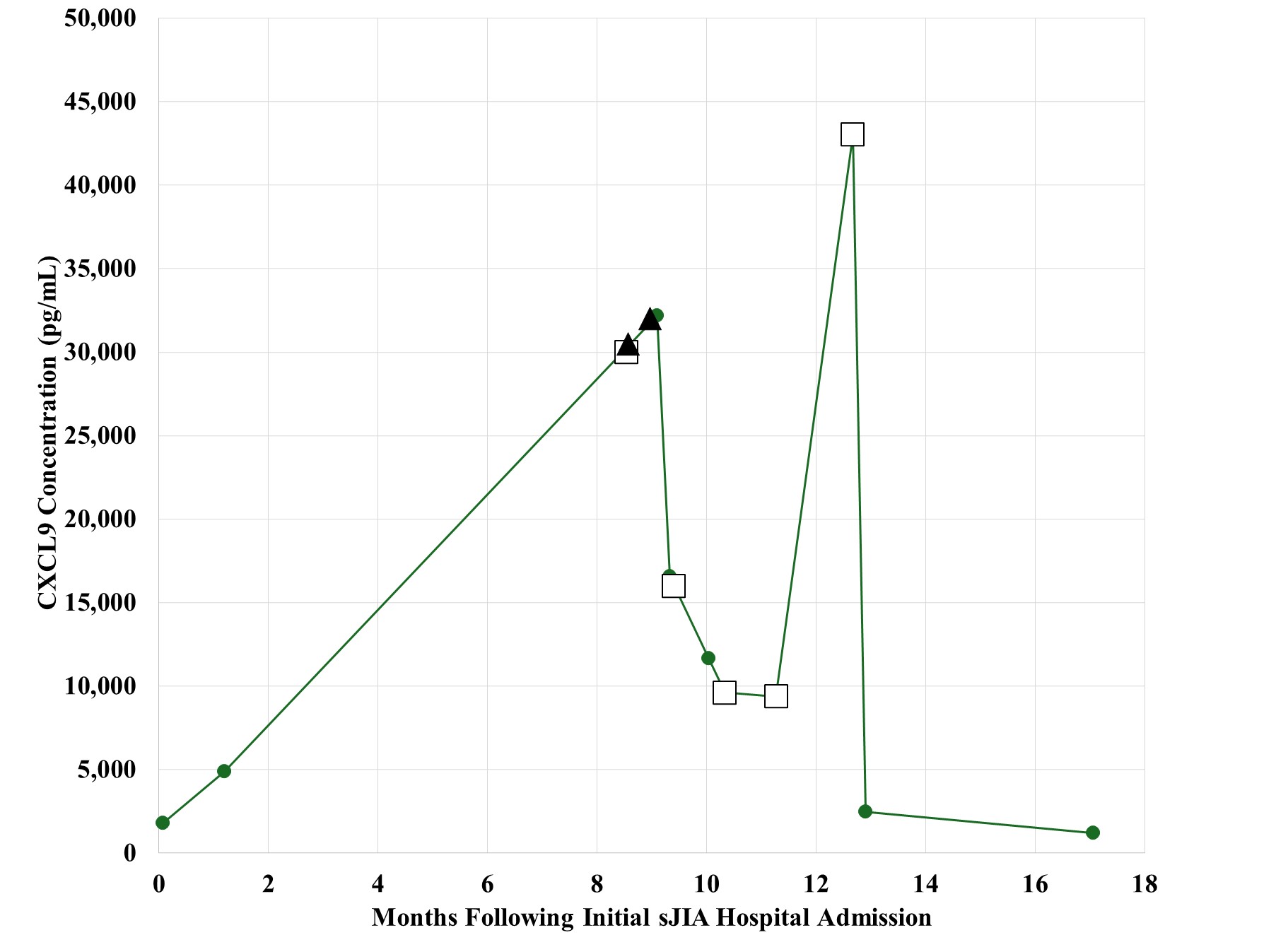

Supplement: Supplementary file 1 [file Image1.jpeg]
