# Supplementary material for: Case Report: Treatment of refractory lung disease in systemic juvenile idiopathic arthritis with cyclophosphamide and rituximab combination therapy
Source: Front Immunol. 2026 May 4;17:1798455. doi: 10.3389/fimmu.2026.1798455 (PMC13180542; doi:10.3389/fimmu.2026.1798455)
Supplement: Supplementary file 2 [file Table1.docx]

| **Name** | **Units** | **Reference**  **Range** | **Measurement Method** |
| --- | --- | --- | --- |
| CRP concentration | mg/dL | 0-0.50 | latex-based immunoassay with immunoturbidimetry |
| CXCL9 concentration | pg/mL | ≤647 | automated microfluidics immunoassay |
| ESR | mm/hr | 0-15 | Westergren procedure |
| Ferritin concentration | ng/mL | 13.7-78.8 | chemiluminescent microparticle immunoassay |
| IL-18 concentration | pg/mL | ≤477 | automated microfluidics immunoassay |
| KL-6 concentration | U/mL | 0-500 | quantitative immunoturbidimetry |
| sIL-2Rα (sCD25) | pg/mL | 175.3-858.2 | quantitative multiplex bead assay |

**Supplementary Table 1**: Inflammatory and disease biomarker reference ranges and laboratory measurement methods. *CRP, C-reactive protein; CXCL9, chemokine (C-X-C motif) ligand 9; ESR, erythrocyte sedimentation rate; IL-18, interleukin-18; KL-6, Krebs von den Lungen 6; sCD25, soluble cluster of differentiation 25; sIL-2Rα, soluble interleukin-2 receptor alpha (chain)
