# Supplementary material for: Case Report: Treatment of refractory lung disease in systemic juvenile idiopathic arthritis with cyclophosphamide and rituximab combination therapy
Source: Front Immunol. 2026 May 4;17:1798455. doi: 10.3389/fimmu.2026.1798455 (PMC13180542; doi:10.3389/fimmu.2026.1798455)
Supplement: Supplementary file 3 [file Table2.docx]

**(A)**

| **Age** | **Cyclophosphamide dose**  **(mg/m^2^)** | **Rituximab dose**  **(mg/m^2^)** |
| --- | --- | --- |
| 12 years, 2 months, 0 days | 750 |  |
| 12 years, 2 months, 1 day |  | 750 |
| 12 years, 2 months, 13 days |  | 750 |
| 12 years, 2 months, 26 days | 850 |  |
| 12 years, 3 months, 26 days | 850 |  |
| 12 years, 4 months, 23 days | 750 |  |
| 12 years, 6 months, 4 days | 750 |  |
| **Cumulative Dose (mg/m^2^)** | **3950** |  |
| **Cumulative Dose (mg)** | **5175** |  |
| **Cumulative Dose (g)** | **5.175** |  |

**(B)**

| **Age** | **Cyclophosphamide dose**  **(mg/m^2^)** | **Rituximab dose**  **(mg/m^2^)** |
| --- | --- | --- |
| 3 years, 10 months, 21 days | 500 |  |
| 3 years, 10 months, 23 days |  | 750 |
| 3 years, 11 months, 11 days |  | 750 |
| 3 years, 11 months, 25 days | 500 |  |
| 4 years, 0 months, 30 days | 500 |  |
| 4 years, 1 month, 27 days | 500 |  |
| 4 years, 3 months, 29 days | 700 |  |
| 4 years, 4 months, 26 days | 700 |  |
| 4 years, 6 months, 20 days | 700 | 750 |
| 4 years, 8 months, 11 days |  | 750 |
| 5 years, 6 months, 21 days | 700 |  |
| 5 years, 6 months, 22 days |  | 700 |
| 5 years, 7 months, 26 days |  | 700 |
| 6 years, 11 months, 24 days |  | 700 |
| 7 years, 1 month, 5 days |  | 700 |
| 8 years, 1 month, 2 days |  | 700 |
| 8 years, 1 month, 16 days |  | 700 |
| **Cumulative Dose (mg/m^2^)** | **4800** |  |
| **Cumulative Dose (mg)** | **2750** |  |
| **Cumulative Dose (g)** | **2.750** |  |

**Supplementary Table 2**: Cyclophosphamide and rituximab doses with corresponding ages. (A) Patient 1. (B) Patient 2.
