# Supplementary material for: Case Report: Treatment of refractory lung disease in systemic juvenile idiopathic arthritis with cyclophosphamide and rituximab combination therapy
Source: Front Immunol. 2026 May 4;17:1798455. doi: 10.3389/fimmu.2026.1798455 (PMC13180542; doi:10.3389/fimmu.2026.1798455)
Supplement: Supplementary file 4 [file Table3.docx]

**(A)**

| **Months Following Initial sJIA Hospital Admission** | **Ferritin Concentration (ng/mL)** | **CXCL9 Concentration (pg/mL)** | **IL-18 Concentration (pg/mL)** | **KL-6 Concentration (U/mL)** | **sIL-2Rα Concentration (pg/mL)** |
| --- | --- | --- | --- | --- | --- |
| 0.00 | 14150.8 |  |  |  |  |
| 0.03 |  |  |  |  |  |
| 0.07 | 10270.9 | 1796 | 144738 | 774 | 2517.9 |
| 0.10 | 11311.2 |  |  |  |  |
| 0.13 | 9100.7 |  |  |  |  |
| 0.17 | 6739.1 |  |  |  |  |
| 1.20 | 3563.1 | 4894 | 131901 | 1427 | 4367.6 |
| 3.07 | 4451.3 |  |  | 2072 |  |
| 6.10 |  |  |  | 1863 |  |
| 7.60 | 5981.1 |  |  |  |  |
| 7.63 | 5290.9 |  |  | 1741 |  |
| 8.20 | 8718.6 |  |  |  |  |
| 8.23 |  |  |  | 2991 | 1231.2 |
| 8.27 | 8551.6 |  |  |  |  |
| 8.33 | 5360.2 |  |  |  |  |
| 8.40 | 13150.2 |  |  |  |  |
| 8.43 | 17243.9 |  |  |  |  |
| 8.47 | 13653.0 |  |  |  |  |
| 8.87 | 38219.9 |  |  |  |  |
| 8.90 | 48228.1 |  |  |  |  |
| 8.97 | 61121.5 |  |  |  |  |
| 9.00 | 54583.8 |  |  |  |  |
| 9.03 | 89765.6 |  |  |  |  |
| 9.07 | 97299.5 |  |  |  |  |
| 9.10 | 79859.9 | 32198 |  | 3087 |  |
| 9.13 | 38128.9 |  |  |  |  |
| 9.17 | 14810.6 |  |  |  |  |
| 9.20 | 15518.2 |  |  |  |  |
| 9.23 | 11192.6 |  |  |  |  |
| 9.27 | 9613.7 |  |  |  |  |
| 9.30 | 9076.4 |  |  |  |  |
| 9.33 | 9374.3 | 16568 |  |  |  |
| 9.37 | 8077.9 |  |  |  |  |
| 9.40 | 8504.2 |  |  |  |  |
| 9.83 | 11025.4 |  |  |  |  |
| 9.93 | 7061.8 |  |  |  |  |
| 9.97 | 4918.5 |  |  |  |  |
| 10.00 | 4657.8 |  |  |  |  |
| 10.03 | 4271.0 | 11639 | 113055 |  |  |
| 10.07 | 4687.9 |  |  |  |  |
| 10.10 | 5011.6 |  |  |  |  |
| 10.13 | 4253.7 |  |  | 3803 |  |
| 10.17 | 4545.7 |  |  |  |  |
| 10.33 | 1884.5 | 9600 |  | 3467 | 795.9 |
| 11.27 | 3313.8 | 9371 |  | 4334 | 1179.9 |
| 12.67 | 8847.3 | 43021 |  | 3547 | 783.0 |
| 12.90 | 303.4 | 2454 | 47133 |  |  |
| 15.07 | 58.8 |  | 27163 |  |  |
| 16.00 | 32.4 |  | 20676 | 2479 |  |
| 17.07 | 22.5 | 1184 | 13077 | 2150 | 482.7 |
| 17.77 | 32.2 |  |  | 2141 |  |

**(B)**

| **Years of Life** | **Ferritin Concentration (ng/mL)** | **ESR (mm/hr)** | **CRP Concentration (mg/dL)** |
| --- | --- | --- | --- |
| 0.89 | 2889.0 |  |  |
| 0.90 |  | 99 | 16.93 |
| 0.91 |  | 111 | 19.49 |
| 0.92 |  | >140 | 13.93 |
| 0.94 |  | 111 | 12.16 |
| 0.95 | 502.0 |  |  |
| 1.00 |  | 97 | 13.19 |
| 1.21 |  | 97 |  |
| 1.31 |  | 99 |  |
| 1.54 |  | 91 |  |
| 1.55 |  | 17 | 5.89 |
| 1.56 | 838.0 |  |  |
| 1.73 |  | 29 | 1.75 |
| 1.91 |  | 32 |  |
| 2.22 | 622.0 | 51 | 1.90 |
| 2.52 |  | 55 |  |
| 2.59 |  | 94 | 12.91 |
| 2.98 |  | 27 |  |
| 3.33 |  | 64 |  |
| 3.56 |  | 92 |  |
| 3.76 | 294.0 | 60 |  |
| 3.85 |  | 15 | 2.51 |
| 3.86 | 51.0 | 6 | 1.11 |
| 3.89 | 65.0 | 12 | 1.48 |
| 3.90 |  | 23 | 0.51 |
| 3.95 |  | 16 | 1.05 |
| 3.95 | 866.0 |  |  |
| 3.96 |  | 18 | <0.30 |
| 3.99 |  |  | <0.30 |
| 4.08 |  | 6 | <0.30 |
| 4.16 |  | 15 | <0.30 |
| 4.33 |  | 15 | <0.30 |
| 4.56 | 47.0 | 26 | 1.00 |
| 4.70 |  | 17 | <0.30 |
| 4.77 |  | 10 | <0.50 |
| 5.33 |  |  | 0.70 |
| 5.51 |  | 23 | 0.54 |
| 5.56 |  | 17 | <0.50 |
| 5.68 |  | 10 | <0.50 |
| 6.23 |  | 4 | <0.50 |
| 6.57 |  | 15 | <0.50 |
| 6.92 |  | 42 | <0.50 |
| 6.98 |  | 17 | <0.50 |
| 7.10 |  | 11 | <0.50 |
| 7.28 |  | 9 | <0.50 |
| 7.86 |  | 13 |  |
| 8.09 |  | 15 | <0.50 |
| 8.13 |  | 9 | <0.50 |
| 9.09 | 39.0 | 9 | <0.50 |
| 9.98 | 39.0 | 8 | <0.50 |
| 10.75 | 118.0 | 42 | 19.16 |
| 11.77 | 55.0 | 31 | 0.52 |
| 12.11 | 42.0 | 15 | <0.50 |
| 12.82 | 45.0 | 17 | <0.50 |
| 13.26 | 45.0 | 16 | <0.50 |
| 14.18 | 82.6 | 31 | 0.29 |
| 14.22 | 76.1 | 18 | 0.10 |
| 14.66 | 64.1 | 20 | 0.30 |
| 15.03 | 64.5 | 23 | 0.18 |
| 18.17 | 68.3 | 34 | 0.19 |

**Supplementary Table 3**: Disease and inflammatory markers over time. (A) Patient 1. (B) Patient 2. *CRP, C-reactive protein; CXCL9, chemokine (C-X-C motif) ligand 9; ESR, erythrocyte sedimentation rate; IL-18, interleukin-18; KL-6, Krebs von den Lungen 6; sIL-2Rα, soluble interleukin-2 receptor alpha (chain).
